# Impact of Weight Loss Strategies on Obesity‐Induced DNA Damage

**DOI:** 10.1002/mnfr.201900045

**Published:** 2019-06-14

**Authors:** Tahereh Setayesh, Miroslav Mišík, Sabine A. S. Langie, Roger Godschalk, Monika Waldherr, Thomas Bauer, Sabine Leitner, Christoph Bichler, Gerhard Prager, Georg Krupitza, Alexander Haslberger, Siegfried Knasmüller

**Affiliations:** ^1^ Department of Internal Medicine I Institute of Cancer Research Medical University of Vienna Vienna Austria; ^2^ VITO‐Health Mol Belgium; ^3^ Centre for Environmental Sciences Hasselt University Hasselt Belgium; ^4^ Department of Pharmacology & Toxicology School for Nutrition Toxicology and Metabolism (NUTRIM) Maastricht University Maastricht The Netherlands; ^5^ Department of Biomedical Imaging and Image‐guided Therapy Medical University of Vienna Vienna Austria; ^6^ Section of Endocrine Surgery Division of General Surgery Department of Surgery Medical University of Vienna Vienna Austria; ^7^ Clinical Institute of Pathology Medical University of Vienna Vienna Austria; ^8^ Department of Nutritional Sciences University of Vienna Vienna Austria

**Keywords:** DNA damage, DNA repair, inflammation, weight loss, Western diet

## Abstract

**Scope:**

Obesity causes DNA damage, which is causally related to several disorders including cancer, infertility, and cognitive dysfunctions. The aim of this study is to investigate whether weight loss improves the integrity of the genetic material.

**Methods and Results:**

Overweight mice are fed ad libitum either with a Western diet (WD), with a 40% caloric restricted WD, or with a high carbohydrate low protein (HCLP) diet. Caloric restriction and also the HCLP diet lead to ca. 30% weight loss, which is paralleled by decreased DNA damage (“comet” formation) and oxidative damage of purines in inner organs, additionally the activity of nucleotide excision repair increased. The effects are more pronounced in animals that have received the HCLP chow. Results of biochemical analyses indicate that the reduction of DNA damage is associated with a decrease of pro‐inflammatory cytokines and lower insulin levels.

**Conclusion:**

The study indicates that weight loss may prevent obesity‐associated adverse health effects due to reduction of overall DNA damage.

## 1 Introduction

Overweight and obesity increased worldwide about sixfold since 1976.^1^ It was stressed by Cancer Research UK that the cancer risks caused by excess body weight will soon eclipse those of tobacco smoking.^2^ Increased BMI values are associated with elevated prevalence of cardiovascular diseases, cancer, and probably also with infertility, cognitive dysfunctions, and reduced life span.^3^, ^4^, ^5^, ^6^

Recently, we showed that a high‐fat diet leads to increased body weight in mice and found evidence for induction of DNA damage in a variety of inner organs.^7^ The aim of the present study was to investigate if weight reduction improves the integrity of the genetic material. DNA damage is one of the most important hallmarks of cancer^8^ and plays also a role in the etiology of infertility and neurological dysfunctions.^3^, ^9^, ^10^ Many earlier studies on nutrition related cancer prevention focused on the antimutagenic properties of specific human foods.^11^ A recent evaluation of the current state of knowledge indicated that also weight loss may improve the integrity of the genetic material.^12^ However, earlier findings with humans are partly controversial and no firm conclusions can be drawn, since consumption of vitamins and minerals, which may have an impact on DNA damage,^13^, ^14^, ^15^ was not taken into consideration.^12^ The findings of animal studies were more consistent but also in these investigations the intake of micronutrients was neglected.

Two common strategies to reduce excessive body weight are i) decreased food consumption without alteration of the overall composition of the diet and ii) changes in the amounts of macronutrients. It was found that high‐protein diets cause weight loss^16^ but long‐term consumption may cause adverse effects including an increase of mortality and cancer.^17^, ^18^, ^19^ Weight loss by consumption of a high‐carbohydrate low‐protein diet (HCLP) is more promising; it was reported that it improved metabolic health parameters and reduced obesity induced inflammation.^18^, ^20^, ^21^

In the present study, we used a mouse model, which reflects obesity and the metabolic syndrome in humans,^22^, ^23^, ^24^ to compare the impact of weight loss by either reduced consumption of a WD or by an HCLP diet on DNA integrity in various inner organs. The diets were composed in such a way that the animals in the different feeding groups received identical amounts of vitamins and minerals.

DNA damage was measured in single cell gel electrophoresis (SCGE) or “comet” assays that are based on the determination of DNA migration in an electric field and are increasingly used.^25^ The migration of damaged genetic material leads to “comets” reflecting the formation of single and double strand breaks and apurininc sites (see Figure S1, Supporting Information).^26^ Since it was postulated that glucose, insulin levels, and fatty acid metabolism may play a key role in obesity‐induced DNA damage,^12^ we measured these parameters in the different feeding groups.

To elucidate the molecular mechanisms by which weight loss affects genomic integrity, we measured alterations of the formation of oxidized DNA bases and of the activity of nucleotide excision repair (NER) in various organs in the weight loss groups and compared the results with those obtained with obese animals. A modified version of the SCGE technique was used to assess the oxidation of purines as it has been postulated that obesity leads to inflammation, and as a consequence, to formation of reactive oxygen species causing damage of DNA bases.^27^, ^28^ To test whether reduction of oxidative DNA damage caused by weight loss is associated with alterations of the inflammation status, we monitored the levels of various pro‐inflammatory cytokines (IL‐6, TNF‐α, monocyte chemoattractant protein‐1 [MCP‐1] and leptin) and of the anti‐inflammatory cytokine adiponectin in plasma. It was postulated that increased body weight affects DNA repair systems via different molecular mechanisms (for review see ref. [12]), in particular NER, which is one of the most important repair system in eukaryotic organisms.^29^ Therefore, we monitored the impact of weight loss on the activity of this repair pathway in different inner organs in obese mice and in the weight loss groups.

## 2 Experimental Section

### 2.1 Animals and Feeding Scheme

The animal study was approved by the Ethics Committee of the Medical University of Vienna (BMWFW‐66.009/0329‐WF/V/3b/2014). Male and female C57BL6/J mice (6 weeks old; *n* = 36, Harland Laboratory, Italy) were housed at the animal facility of the Institute of Cancer Research, Medical University of Vienna. The animals were maintained at 22 ± 2 °C under a 12:12 h light–dark cycle.

**Figure 1:**
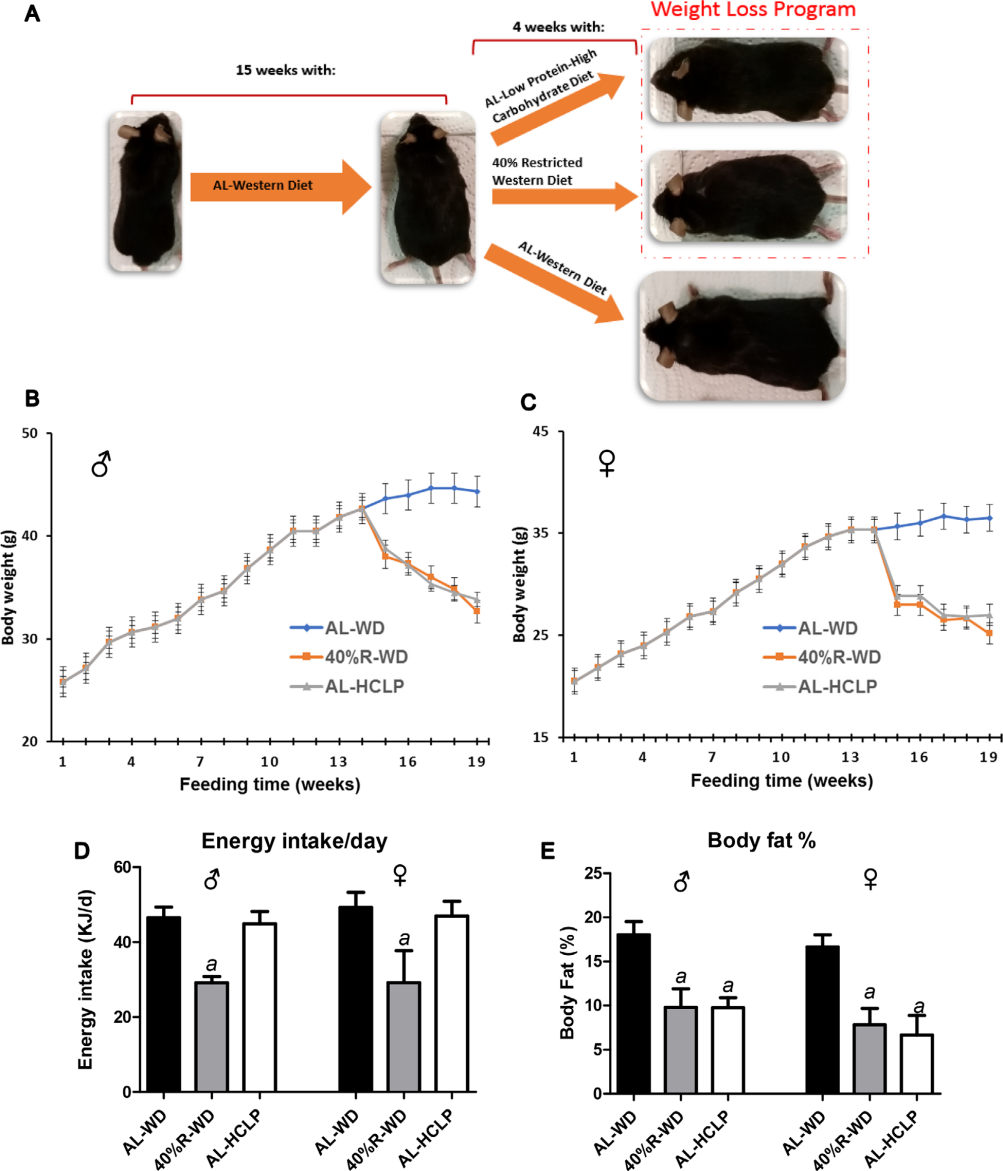
Effect of weight loss after consumption of a 40%‐R‐WD or a AL‐HCLP diet on obese animals that received a Western Diet (WD) for 14 weeks. Schematic design of the study A), impact of consumption of the different diets on body weights B and C), percentage of body fat in mice at the end of the experiment D), and energy intake (KJ) per day for each group (E). Bars show mean ± SD (with six animals per group). (***a***) indicates a statistically significant difference (*p *≤ 0.05, Bonferroni's method).

After acclimatization for 1 week, the animals were fed ad libitum with a WD (AL‐WD), which reflects food consumption in industrialized Western countries.^30^ After 14 weeks, the mice were divided into three subgroups (six males and six females per group) and were fed with the AL‐WD, with a 40% restricted WD (40%‐R‐WD) or with the AL‐HCLP (**Figure** 1A).

The composition of the diets is described in Table S1, Supporting Information. It was found in earlier studies that micronutrients affected the stability of the genetic material.^13^, ^15^ To exclude bias of vitamin and mineral consumption on the results, their contents in the diets were designed in such a way that the consumption levels in the feeding groups were identical (see Table S1, Supporting Information). The feeding scheme was designed on the basis of a preliminary study; the animals that received the 40%‐R‐WD and the AL‐HCLP diet had identical weights at the end of the feeding period.

After 19 weeks, the mice were sacrificed by cervical dislocation. Subsequently, blood and inner organs (i.e., liver, colon, brain, testes, and ovaries) were collected and used for the preparation of slides for SCGE experiments (standard conditions and determination of Fpg sensitive sites). Part of the samples was frozen immediately after the collection and stored in liquid nitrogen for NER experiments and biochemical analyses.

### 2.2 Body Composition

Fat, lean, and fluid mass of mice were measured with a body composition analyzer for a small live animals (EchoMRI‐100H, Houston, TX). The unanesthetized mice were weighed and scanned.

### 2.3 Detection of DNA Damage by SCGE (Standard Conditions)

To determine DNA damage, SCGE experiments were conducted under alkaline conditions according to OECD guideline #489.^31^ Inner organs (i.e., liver, colon, brain, testes, and ovaries) of mice were removed, washed, and homogenized. Cells from the colon mucosa were collected by scraping.^7^ Nuclei were isolated from the tissue homogenates as described in detail by Sasaki et al.^32^ Subsequently, the slides were electrophoresed, then the gels were stained with propidium iodide (Sigma–Aldrich, Germany, 20 µg mL^–1^) and air dried.^7^ All slides were coded and scorers were not aware of the origin of the slides.

### 2.4 Assessment of the Formation of Oxidized Purines

Experiments with the lesion‐specific enzyme formamidopyrimidine DNA glycosylase (Fpg, 500U, New England BioLabs, Austria) were conducted as described earlier.^7^ The activity of the enzyme was measured before the main experiment (data not shown). The extent of DNA migration attributable to Fpg‐sensitive sites was calculated in all experiments by subtraction of the corresponding enzyme buffer values.^33^

### 2.5 Determination of Nucleotide Excision Repair

Nucleotide excision repair (NER) was monitored in different organs with a modified SCGE protocol.^34^, ^35^, ^36^, ^37^ This assay measures the ability of NER‐related enzymes that are present in cell extracts and incise damaged DNA.^36^ Substrate nucleoids were isolated from untreated HeLa cells, which were purchased from the American Tissue Culture Collection (ATCC) and were cultivated in T75 flasks in DMEM growth medium in 37 °C and 5% CO_2_. The cells were exposed to UV (4 J m^−2^ for 3 s) or left untreated (controls). After treatment, the cells were trypsinized (with 1.0% trypsin) at more than 80% confluency, embedded in low melting agarose (0.5, Gibco, Paisley, UK) on glass slides (three gels per experimental point) and lysed overnight (4 °C) with lysis buffer (2.5 m NaCl, 0.1 m EDTA, 0.01 m Tris, 0.25 m NaOH plus 1% Triton X‐100 and 10% DMSO added immediately before use).

To prepare the protein/enzyme extracts, 50 mg frozen tissues (liver, brain, colon, testes, and ovaries) were thawed. After washing with cold PBS, the material was resuspended in buffer A (45 mm HEPES, 0.4 m KCl, 1 mm EDTA, 0.1 mm dithiothreitol, 10% glycerol, adjusted to pH 7.8 using KOH, 100 µL per 50 mg tissue). The resulting suspensions were snap‐frozen and thawed again. Lysis was completed by adding 30 µL 1% Triton X‐100 in buffer A per 100 µL of the extract. Lysates were centrifuged at 15 000 × *g* at 4 °C for 5 min. Tissue extracts were diluted to a concentration of 0.5 mg mL^–1^ (wet weight) and stored at −80 °C overnight. Subsequently, protein extracts were thawed and four volumes of reaction buffer N (45 mm HEPES, 0.25 mm EDTA, 2% glycerol, 0.3 mg mL^–1^ BSA, adjusted to pH 7.8 with KOH) and 2.5 mm ATP were added. Of these suspensions, 50 µL aliquots were incubated with the gel‐embedded UV‐treated nucleoids at 37 °C for 30 min. Alkaline treatment (30 min) and electrophoresis (30 min) were conducted as described for standard comet assays. The induction of DNA breaks leading to increased tail intensities, which is indicative for the NER activity, was monitored. After subtraction of the background levels, the repair capacity was calculated according to Langie et al.^36^

### 2.6 Determination of Comet Formation

From each organ, three gels were prepared per animal for each endpoint and 50 cells were evaluated per gel. Comet formation was analyzed under a fluorescence microscope (Nikon EFD‐3, Tokyo, Japan) at 400× final magnification. DNA migration was determined with a computer‐aided image analysis system (Comet Assay IV, Perceptive Instruments, Bury St Edmunds, UK). The percentage of DNA in the tail (%DNA in tail) was assessed as a parameter of comet formation.

### 2.7 Measurement of Biochemical Parameters

Triglyceride and glucose concentrations were measured in blood with MultiCare strips (Biochemical Systems International, Italy). Plasma insulin was quantified with a mouse insulin ELISA kit (Thermo Fisher Scientific, MA) according to the instructions of the manufacturer. Circulating levels of inflammatory cytokines (TNF‐α, MCP‐1, IL‐6, IL‐1β, leptin, and adiponectin) were monitored in plasma with commercially available LUMINEX kits (Affymetrix, eBioscience, Austria) according to the instructions of the manufacturer.

All samples were analyzed in duplicate.

### 2.8 Statistical Analyses

The percentage of DNA in the comet tails was determined as suggested by Bright.^38^ The medians of %DNA in the tails were computed in each replicate, *t*, subsequently the means of the median %DNA in tail of three replicates were compared. Statistical significance of the results of SCGE experiments was analyzed by the nonparametric Mann–Whitney *U*‐test.

Group differences between terminal body weights, fat composition of the animals, and inflammatory and biochemical markers were evaluated using multifactor ANOVA and multiple‐range test (Bonferroni's method). For all comparisons, results with *p* values ≤ 0.05 were considered significant. The analyses were performed using GraphPad Prism 5.0 (GraphPad Software, San Diego, CA).

## 3 Results

### 3.1 Effects of Different Feeding Schemes on Body Weights

After a feeding period of 14 weeks with the AL‐WD, the average body weights in male and female animals were 43 and 32 g, respectively. The weight loss phase started at week 15, when the animals were switched to either a 40%‐R‐WD or an AL‐HCLP diet (Figure 1A). The body weights of the animals in the 40%‐R‐WD group and in the AL‐HCLP group decreased continuously in the following weeks (for details see Figures 1B,C). At week 19, the weights of the animals were in both groups almost identical (40%‐R‐WD: males 32 g and females 25 g; AL‐HCLP: males 33 g and females 26 g). Mice, which had received the AL‐WD, had substantially higher body weights (males 47 g; females 37 g).

The weight loss of the two intervention groups was paralleled by a pronounced decrease of body fat. After consumption of the 40%‐R‐WD diet and of the HCLP diet, the mice lost ≈45% of the overall body fat at the end of the weight loss phase (Figure 1D), whereas energy intake was only reduced in the 40%‐R‐WD group (Figure 1E).

### 3.2 Impact of Weight Loss on Overall DNA Integrity

**Figure 2:**
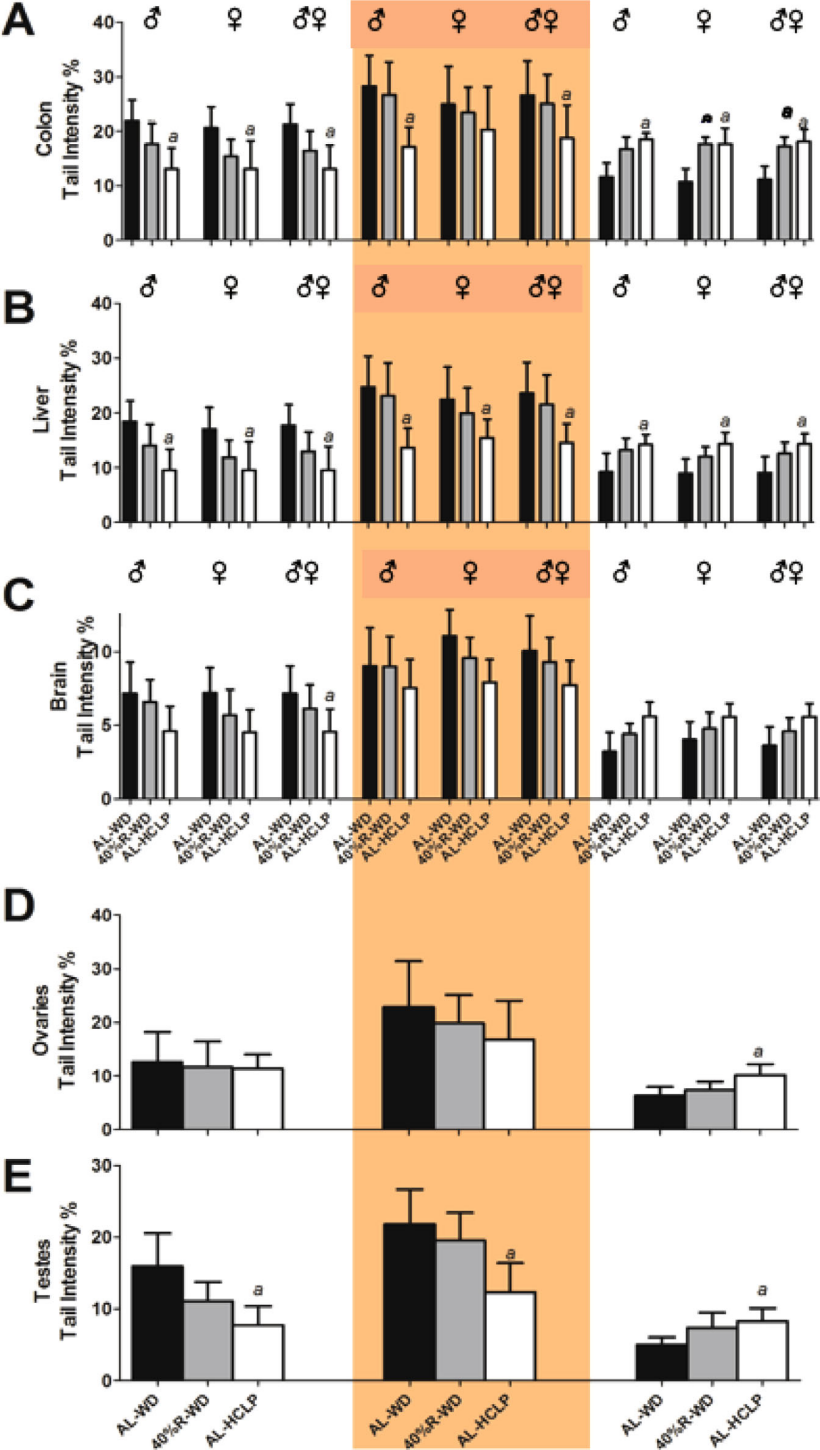
Impact of the different diets on DNA damage, formation of oxidized purines (Fpg‐ specific sites), and NER activity in different inner organs of mice. The feeding scheme is shown in Figure 1A. The left parts of the graphs show the effects of WD feeding and weight loss on DNA damage. The middle and right sections show the effects of weight loss on net‐Fpg sensitive sites and on the NER incision activity in different organs. From each organ, three slides were made and 50 cells were analyzed per slide. Formation of oxidized purines was assessed by treatment of nuclei with formamidopyrimidine DNA glycosylase (Fpg). Bars show values obtained with the enzyme after subtraction of results obtained with the respective buffers. The final NER incision activity was calculated after subtracting the background levels. Bars show mean of the medians ± SD of six animals per group. (***a***) indicates a statistically significant difference (*p *≤ 0.05, nonparametric Mann–Whitney *U*‐test).

**Figure** 2A–E shows that weight loss improved the DNA integrity in a variety of inner organs of the obese animals. We found a clear tendency toward reduced DNA damage (assessed as strand breaks in SCGE experiments under standard conditions) in colon, liver, brain, and testes after reduced consumption of the WD (Figure 2, left section). However, none of these effects was significant. In contrast, a clear decrease of DNA damage was observed after weight loss with the AL‐HCLP chow in colon, liver, and testes. The effects in male and female animals were almost identical.

A similar pattern was found in experiments that were performed to assess the formation of oxidized purines (Figure 2, middle section). A moderate decrease of Fpg‐sensitive sites was observed in the 40%‐R‐WD group; whereas in the AL‐HCLP group, the effects were more pronounced and significant in colon, liver, testes, and brain.

The right sections of Figure 2 show alterations of the NER incision activity after weight loss. The repair capacity increased significantly in several organs (liver, colon, testes, and ovaries) in the AL‐HCLP diet group. A similar effect (which did not reach significance) was seen in the brain. Again, the effects were less pronounced in the 40%‐R‐WD group. The overall repair capacity decreased in all feeding groups in the following order: colon > testes, liver, and ovary > brain.

No pronounced gender specific effects were seen regarding the DNA integrity in SCGE assays under standard conditions and also in Fpg and NER experiments.

### 3.3 Impact of Weight Loss on Metabolic Health Parameters

**Figure 3:**
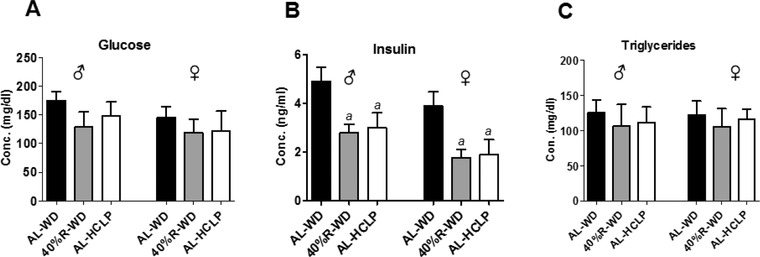
Impact of the different diets on glucose, insulin and triglyceride levels in plasma. The animals were either fed continuously ad libitum with the WD, with the 40%‐R‐WD or with the AL‐HCLP. Bars indicate mean ± SD of six animals per group. (***a***) indicates a statistically significant difference (*p *≤ 0.05, Bonferroni's method).

**Figure** 3A–C shows alterations of the glucose, insulin and triglyceride levels after weight loss. Glucose concentrations in the blood were reduced in both sexes in the range between 15% and 26% (Figure 3A). However, these effects did not reach significance. On the contrary, clear reduction of the insulin levels was observed with both dietary regimens, while the triglyceride concentrations were in all experimental groups similar (Figure 3B,C).

### 3.4 Alterations of the Levels of Pro/Anti‐Inflammatory Cytokines after Weight Loss

**Figure 4:**
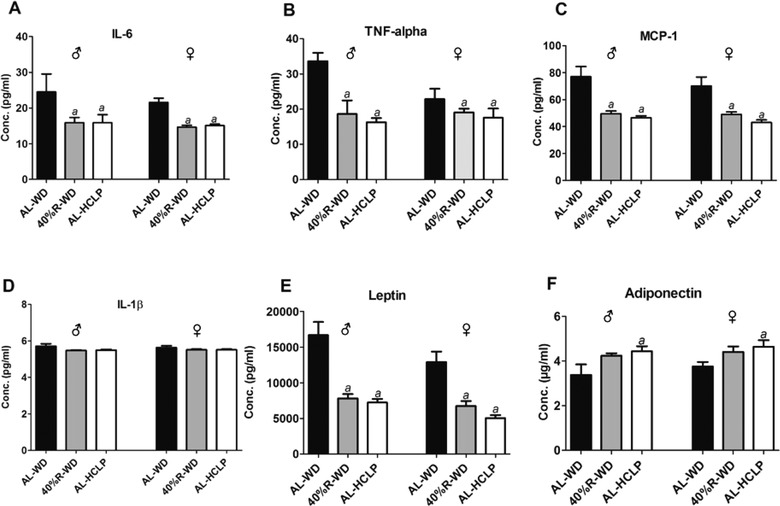
Impact of the different diets on the levels of inflammatory cytokines in plasma. The animals were either fed continuously with the AL‐WD or for 5 weeks with a 40%‐R‐WD or with the AL‐HCLP. Bars indicate mean ± SD that were obtained from six animals per group. (***a***) indicates statistical significance (*p *≤ 0.05, Bonferroni's method).

**Figure** 4 summarizes the results of measurements of various inflammatory markers in the plasma of the different feeding groups. Both weight loss diets caused a pronounced decrease of pro‐inflammatory markers (IL‐6, MCP‐1, leptin, and TNF‐α) while the IL‐1β remained unchanged (Figure 4A–E). Plasma adiponectin levels increased, again the most pronounced effect was seen in the AL‐HCLP animals (Figure 4F).

## 4 Discussion

The aim of this study was to investigate whether weight loss improves the integrity of the DNA, which plays an important role in the etiology of various diseases that are associated with increased body mass (e.g., cancer, infertility, cognitive dysfunctions). Weight reduction was either achieved via restricted consumption of a Western diet (by 40%), which reflects the uptake of macronutrients in industrialized Western countries,^39^ or by feeding an HCLP chow.

The study was designed in such a way that the weights of the animals in the calorie restriction group (40%‐R‐WD) and in the AL‐HCLP group were identical at the end of the feeding period. Although calorie intake in the latter group was higher than in the restriction group, the body weights and the percentage in body fat declined in a similar manner (see Figure 1B–D). Also in an earlier study by Lee et al.,^20^ pronounced weight loss was observed in mice after a switch from a high‐fat diet to a high‐carbohydrate diet. Furthermore, the weight gain that was found after ad libitum feeding of isocaloric high‐fat and of high‐carbohydrate diets was less pronounced with the latter chow.^40^, ^41^ A possible reason for this phenomenon may be that carbohydrates contributed to a lesser extent as fats to de novo lipogenesis.^42^

We found that the decrease of the body weights (by ca. 30 %) as a consequence of reduced consumption of the WD caused a moderate (not significant) decline of DNA damage in various inner organs. More pronounced effects were consistently observed after weight loss with the AL‐HCLP chow (Figure 2, left sections). The latter effects reached significance in colon, liver, and testes, but not in brain and ovaries. Interestingly, brain and ovaries were also the organs with the lowest overall DNA repair activities. Both organs have a low cell turn over, which may be an important determinant for DNA damage and repair.^43^ As mentioned above, findings of earlier human studies concerning the association between obesity and DNA damage are in general inconsistent, possibly due to the fact that they were not controlled in regard to the consumption of vitamins, minerals, and other potential DNA protective factors such as secondary plant constituents, which affect the stability of the genetic material.^12^, ^13^, ^14^, ^15^ However, results of animal studies that were conducted under more controlled conditions generally indicated a correlation between DNA damage and overweight (for review see ref. [12])

A recent evaluation of the literature showed that multiple mechanisms (such as impaired DNA repair, reduction of telomere lengths, inflammation, formation of reactive oxygen species, hormonal effects, and also formation of advanced glycation end products) could lead to DNA damage as a consequence of increased body weight (for details see ref. [12]). However, experimental evidence for relations between these processes and DNA damage is scarce. Notably, several findings point toward an association between formation of ROS and damage of the genetic material (for review see ref. [12]).

The reduction of Fpg sensitive lesions (Figure 2, middle sections) indicated that (at least part) of the effects that were seen in the SCGE‐experiments is due to oxidative damage of DNA bases. This observation is in agreement with results of a study in which mice were fed with a high‐fat diet (HFD) or with a standard chow.^7^ Furthermore, higher levels of 8‐OHdG were detected in some (not all) obesity studies with rodents, while human studies yielded strongly conflicting results possibly as a consequence of poor study designs (for details see ref. [12]). The assumption that oxidative stress has a marked impact on DNA stability in obese individuals (which could lead to malignant transformation) is also supported by the conclusions reached in a recent review of Usman and Volpi.^44^ Furthermore, a number of human investigations were published recently indicating that weight loss reduces obesity associated oxidative stress (for details see ref. [45]).

Increased NER activity was observed in several organs (liver, colon, ovaries, and testes) after weight loss indicating that impairment of repair processes contributed to genomic instability in obese animals. Consistently, higher activities were seen in the AL‐HCLP group compared to the 40%‐R‐WD group (Figure 2, right segment). It is known that several repair functions decline as a consequence of increased body weight and different molecular mechanisms including alterations of the p53 status, reduction of telomere lengths as well as hormonal effects were postulated to play a causal role (for review see ref. [12]). It was reported that the NER levels in peripheral lymphocytes of humans are inversely related to the BMI.^29^ Many investigations indicate that NER proteins play an important role in the regulation of cellular responses to oxidative DNA damage.^46^ Therefore, it is conceivable that the reduced activity of this repair system is causally related to oxidation of DNA bases in obese animals. Himbert et al.^45^ evaluated the literature regarding effects of weight loss on DNA repair functions and telomere lengths. The authors concluded that it is not clear at present whether the reduction of the body weight affects the repair capacity in humans in general and increases the telomere lengths.

The measurement of metabolic and inflammation parameters, which were performed in the present study, provide a potential explanation for the mechanisms by which weight loss improved the integrity of the genetic material via reduction of oxidative DNA damage. The insulin levels decreased significantly in both weight loss groups (Figure 3) and former in vitro studies demonstrated that this hormone causes formation of ROS and, in consequence, generated DNA damage.^47^, ^48^ This observation was confirmed in subsequent animal experiments.^47^ In addition, reduction of inflammation may play a crucial role in the prevention of DNA damage by weight loss. As mentioned in the Introduction section, obesity is characterized by oxidative stress via production of cytokines. Figure 4A–F shows that the levels of inflammatory cytokines (IL‐6, TNF‐α, MCP‐1, and leptin) decreased as a consequence of weight loss while the concentration of adiponectin (which has anti‐inflammatory properties) increased. Notably, the levels of leptin declined in the order AL‐WD→40%‐R‐WD→AL‐HCLP (Figure 4) and the same order was observed in SCGE measurements (under standard conditions, as well as in Fpg and NER experiments), while an inverse association was seen between DNA damage and adiponectin concentrations.

Notably, no sex‐specific differences were found in the present experiments, suggesting that sex hormones do not play a crucial role in obesity‐related DNA damage, oxidative base damage, and NER. This is an important observation since obesity leads to increased production of estrogens that can cause DNA damage via increased mitotic activity and/or directly via formation of DNA reactive metabolites (for review see ref. [12]).

As described above, we found more pronounced reduction of DNA damage and oxidative base damage as well as higher NER activities in the AL‐HCLP group than in the group that received reduced amounts (40%) of the WD. The reasons for this phenomenon are unclear. Possibly, differences in protein consumption (15 % in the AL‐WD and 5 % in the AL‐HCLP diet) caused this phenomenon. Recently, it was reported that the rates of micronuclei (reflecting structural and numerical chromosomal aberrations) in lymphocytes are increased in humans upon regular consumption of protein‐rich foods.^17^

The reduction of DNA damage after weight loss may be indicative of a decrease of overweight‐associated cancer risks in different organs. It is well documented that liver and colon are important targets for induction of tumors in obese individuals.^49^ In the liver, endoplasmic reticulum stress, which causes cell death and increased compensatory cell proliferation, was postulated to promote the development of hepatocellular carcinoma.^49^ The induction of colorectal cancer is possibly the result of secretion of pro‐inflammatory cytokines due to increased permeability of the intestinal mucosa as a consequence of excess body fat (for details see ref. [49]).

The effects of weight loss on DNA stability in the brain are of interest with regard to neurological disorders and cognitive dysfunctions as a consequence of obesity. The latter issue was addressed in a review by Prickett et al.^4^ The authors stated that the evaluation of the literature suggested a clear association between cognitive dysfunctions and overweight in adults. The role of DNA damage is at present not fully understood, but evidence is increasing that impaired DNA repair plays a causal role in Alzheimer's and Parkinson's disease. Furthermore, it was postulated that oxidative DNA damage (which was reduced in the present study as a consequence of weight loss in the HCLP group), could decrease cognitive functions with age.^10^

## 5 Conclusions

We showed for the first time in an animal study that was controlled with regard to consumption of micronutrients (that may have an influence on the results) that weight loss caused reduction of DNA damage in multiple inner organs. Furthermore, we are the first who showed that reduced formation of comets (reflecting single‐, double‐strand breaks and apurinic sites) in SCGE experiments was associated with decreased formation of oxidized DNA bases and increased activity of NER. It is possible that the decline of the insulin levels and of inflammation account for these effects. However, it cannot be excluded that other molecular mechanisms play a relevant role. We found clear evidence that consumption of the AL‐HCLP diet had a more pronounced impact on the integrity of DNA in all organs than reduced intake of the WD. These results are relevant for the development of healthy weight loss strategies.

## Conflict of Interest

The authors declare no conflict of interest.

## Supporting information

Supplementary Information
