# Supplementary material for: Impact of Weight Loss Strategies on Obesity‐Induced DNA Damage
Source: Mol Nutr Food Res. 2019 Jun 14;63(17):1900045. doi: 10.1002/mnfr.201900045 (PMC6771950; doi:10.1002/mnfr.201900045)
Supplement: Supplementary file 1 — Supplementary Information [file MNFR-63-na-s001.doc]

**Supplementary Table 1.** Composition of the diets**.**

| **Ingredients/**  **Formula** | **Western Diet** | **40% restricted Western diet** | **Low Protein High carbohydrate Diet** |
| --- | --- | --- | --- |
|  | **(% kcal)** | **(% kcal)** | **(% kcal)** |
| Protein | 15.2 | 15.2 | 5.0 |
| Carbohydrate | 42.7 | 42.7 | 53.0 |
| Fat | 42.1 | 42.1 | 42.0 |
| Total energy/g diet | 4.5 kcal/g | 4.5 kcal/g | 4.5 kcal/g |
| **Individual component** | **(g/kg)** | **(g/kg)** | **(g/kg)** |
| Casein | 195.0 | 195.0 | 58.0 |
| DL-Methionine | 3.0 | 3.0 | 0.9 |
| Sucrose | 341.0 | 341.0 | 420.0 |
| Corn starch | 150.0 | 150.0 | 150.0 |
| Anhydrous milkfat | 210.0 | 210.0 | 210.0 |
| Mineral mix | 35.0 | 49.0 | 35.0 |
| Calcium carbonate | 4.0 | 4.0 | 1.0 |
| Vitamin mix | 10.0 | 14.0 | 10.0 |
| Cholesterol | 1.5 | 1.5 | 1.5 |
| Ethoxyquin | 0.04 | 0.04 | 0.04 |
| Cellulose | 50.0 | 50.0 | 63.56 |
| Maltodextrin | - | - | 50.0 |
| Calcium Phosphate | - | - | 21.6 |

**Supplementary Figure 1.**


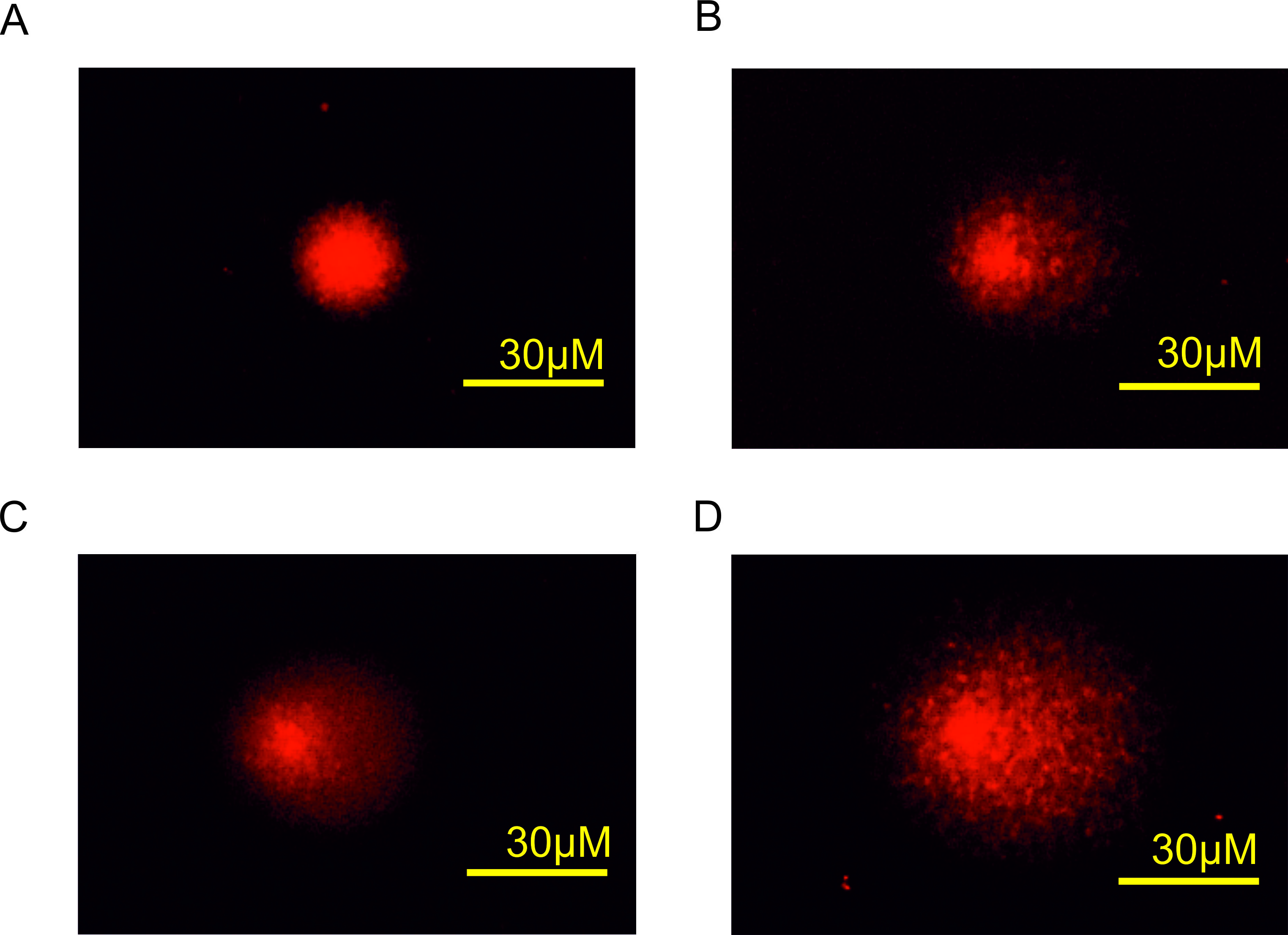


**Supplementary Figure 1.** Photographic images of comets (final magnification 400). Figures A to D show photographic images of comets reflecting different extents of DNA-migration.
